# Suction-Gas Plant v. Boiler Engine

**Published:** 1912-05-11

**Authors:** 


### Suction-Gas Plant v. Boiler Engine.

*To the Editor of* The Hospital.

Sir,—In reply to the letter of Mr. William Kay in your issue of April 27, suction-gas plant is not intended for the purpose of heating buildings. It is intended to furnish gas at a low cost to be used in gas engines, to drive electric generating plant, hydraulic plant, pumps, or anything that may require to be driven. It is of use in country places where there is no gas supply, and is employed for the purposes mentioned above and others.

The suction-gas producer, and the gas engine using suction-producer gas, are also largely taking the place of the steam engine using steam generated in a steam boiler, not only in country places, but even in towns, where steam can be generated under the best conditions, and where there is a supply of town's gas. The reason for its adoption both in country places and in towns is its cheapness. Suction-producer gas plant is guaranteed to consume only 1 lb. of coal per horse-power per hour, where a steam engine and boiler plant will consume from 3 lb. per horse-power upwards. On the other hand, as usual with new apparatus, the suction-gas producer requires a considerable amount of understanding. Only anthracite coal, coke, or charcoal can be employed in the producer furnace. There is an earlier apparatus, the pressure-producer plant, which furnishes gas from any kind of coal, and at as cheap a rate as the suction plant. One great advantage claimed for the suction-gas producer plant is that gas is made as and when it is required, exactly in accordance with the requirements of the engine that is using it. It will be seen from the above, that while the suction-gas producer plant is a very useful servant, it can only be applied to heating purposes, if the gas engine using the gas is employed to generate electricity, and the electric current is employed in electrical heating appliances.—Yours, etc.,

Engineer.

[Owing to the great pressure on our space we have had to hold over several letters this week.]

